# Fast but Not Furious. When Sped Up Bit Rate of Information Drives Rule Induction

**DOI:** 10.3389/fpsyg.2021.661785

**Published:** 2021-11-11

**Authors:** Silvia Radulescu, Areti Kotsolakou, Frank Wijnen, Sergey Avrutin, Ileana Grama

**Affiliations:** ^1^Utrecht Institute of Linguistics-OTS, Utrecht University, Utrecht, Netherlands; ^2^Amsterdam Centre for Language and Communication, Faculty of Humanities, University of Amsterdam, Amsterdam, Netherlands

**Keywords:** rule induction, entropy, channel capacity (information rate), generalization (psychology), category formation, bit rate

## Abstract

The language abilities of young and adult learners range from memorizing specific items to finding statistical regularities between them (*item-bound generalization*) and generalizing rules to novel instances (*category-based generalization*). Both external factors, such as input variability, and internal factors, such as cognitive limitations, have been shown to drive these abilities. However, the exact dynamics between these factors and circumstances under which rule induction emerges remain largely underspecified. Here, we extend our information-theoretic model (Radulescu et al., 2019), based on Shannon’s noisy-channel coding theory, which adds into the “formula” for rule induction the crucial dimension of *time*: the rate of encoding information by a time-sensitive mechanism. The goal of this study is to test the *channel capacity-*based hypothesis of our model: if the *input entropy per second* is higher than the maximum rate of information transmission (bits/second), which is determined by the *channel capacity*, the encoding method moves gradually from *item-bound generalization* to a more efficient *category-based generalization*, so as to avoid exceeding the *channel capacity*. We ran two artificial grammar experiments with adults, in which we sped up the bit rate of information transmission, crucially not by an arbitrary amount but by a factor calculated using the *channel capacity* formula on previous data. We found that increased bit rate of information transmission in a repetition-based XXY grammar drove the tendency of learners toward *category-based generalization*, as predicted by our model. Conversely, we found that increased bit rate of information transmission in complex non-adjacent dependency *aXb* grammar impeded the *item-bound generalization* of the specific *a_b* frames, and led to poorer learning, at least judging by our accuracy assessment method. This finding could show that, since increasing the bit rate of information precipitates a change from *item-bound* to *category-based generalization*, it impedes the *item-bound generalization* of the specific *a_b* frames, and that it facilitates *category-based generalization* both for the intervening *Xs* and possibly for a/b categories. Thus, sped up bit rate does not mean that an unrestrainedly increasing bit rate drives rule induction in any context, or grammar. Rather, it is the specific dynamics between the *input entropy* and the maximum *rate of information transmission*.

## Introduction

Both young and adult learners possess a domain-general distributional learning mechanism for finding statistical patterns in the input (Saffran et al., 1996; Thiessen and Saffran, 2007), and a learning mechanism that allows for category (rule) learning (Marcus et al., 1999; Wonnacott and Newport, 2005; Smith and Wonnacott, 2010; Wonnacott, 2011). While previously cognitive psychology theories claimed that there are two qualitatively different mechanisms, with rule learning relying on encoding linguistic items as abstract categories (Marcus et al., 1999), as opposed to learning statistical regularities between specific items (Saffran et al., 1996), recent views converge on the hypothesis that one mechanism, *statistical learning*, underlies both item-bound learning and rule induction (Aslin and Newport, 2012, 2014; Frost and Monaghan, 2016; Radulescu et al., 2019). Rule induction (generalization or regularization) has often been explained as resulting from processing input variability (quantifiable amount of statistical variation), both in young and adult language learners (Gerken, 2006; Hudson Kam and Chang, 2009; Hudson Kam and Newport, 2009; Reeder et al., 2013).

This study looks into the factors that drive the inductive step from encoding specific items and statistical regularities to inferring abstract rules. While supporting the *single-mechanism hypothesis* and a *gradient of generalization* proposed previously (Aslin and Newport, 2012, 2014), in Radulescu et al. (2019), we took a step further in understanding the two qualitatively different representations discussed in previous research, which we dubbed, in accordance with previous suggestions (Gómez and Gerken, 2000), *item-bound generalizations* and *category-based generalizations.* While *item-bound generalizations* describe relations between specific physical items (e.g., a relation based on physical identity, like “*ba* always follows *ba*” or “*ke* always predicts *mi*”), *category-based generalizations* are operations beyond specific items that describe relationships between categories (variables), e.g., “Y always follows X,” where Y and X are variables taking different values. In order to explain *how* and *why* a single mechanism outputs these two qualitatively different forms of encoding, Radulescu et al. (2019) proposed an information-theoretic model of rule induction as an encoding mechanism. In this model, based on Shannon’s communication theory (1948), we put together both the statistical properties of the input, i.e., *input entropy*, and the finite capacity of the brain to encode the input. In information-theoretic terms at the computational level, in the sense of Marr (1982), we define encoding capacity as *channel capacity*, that is, the finite rate of information transmission (entropy per unit of time, bits/s), which might be supported by certain cognitive capacities, e.g., memory capacity, at the algorithmic level.

Indeed, previous research hinted at cognitive constraints, i.e., memory limitations, on rule learning: the *Less-is-More* hypothesis (Newport, 1990, 2016) proposed that differences in tendency to generalize between young and adult learners stem from maturational differences in memory development: limited memory capacity leads to difficulties in storing and retrieving low-frequency items, which prompts the overuse of more frequent forms leading to overgeneralization. A few studies investigating the nature of these cognitive constraints showed that, while there is some evidence for the *Less-is-More* hypothesis (Hudson Kam and Newport, 2005, 2009; Hudson Kam and Chang, 2009; Wonnacott, 2011), it is not yet clear under *what* specific circumstances and *why* memory constraints should drive rule learning (Perfors, 2012; Hudson Kam, 2019). Cognitive constraints on regularization were also found in nonlinguistic domains (Kareev et al., 1997; Ferdinand et al., 2019), while constrained regularization tendencies were found to be similar across language domains, morphology vs. word order (Saldana et al., 2017).

Nevertheless, the exact cognitive load and mechanisms at stake in rule induction have yet to be thoroughly specified. To this end, Radulescu et al. (2019) offer an extended and more refined information-theoretic approach to the *Less-is-More* hypothesis, by proposing an entropy model for rule induction, which quantifies the specific pattern of statistical variability in the input (i.e., *input entropy*, measured in bits) to which the brain is sensitive, and hypothesizes that rule induction is driven by the interaction between the input entropy and the finite encoding capacity of the brain (i.e., *channel capacity*). Crucially, the model proposes that rule induction is an automatic process that moves *gradually* – *bit by bit* – from a high-fidelity item-specific encoding (*item-bound generalization*) to a more general abstract encoding (*category-based generalization*), as a result of the input entropy being higher than the *channel capacity*, i.e., the maximum rate of information encoding (bits/s). The model is based on Shannon’s *entropy* and noisy-channel coding theory (Shannon, 1948), which says that in a communication system, a message (or information) can be transmitted reliably (i.e., with the least loss in bits of information), if, and only if, encoded using an encoding method that is efficient enough so that the rate of information transmission (i.e., per unit of time), including noise, is below the capacity of the channel. If the rate of information transmission (bit rate) is higher than the *channel capacity*, then another more efficient encoding method can be found, but the *channel capacity* cannot be exceeded.

Based on these concepts, our entropy model for rule induction posits that the change in encoding method, i.e., from *item-bound* to *category-based generalization*, is driven by a kind of a regulatory mechanism, which moves from an inefficient encoding method (with loss of information), to a more efficient encoding method, which allows for higher input entropy to be encoded reliably (with the least loss possible) per second, but crucially below the capacity of the channel. The reliability of encoding should be understood intuitively as given by the least loss of information (caused by noise interference) against the sent message. Thus, this model adds into the rule induction “formula” the crucial dimension of time, i.e., the rate of encoding information by a time-sensitive encoding mechanism, and, consequently, the decrease in loss of information by moving to a more efficient encoding.

A few studies used different (not information-theoretic) methods of quantifying and manipulating a time-dependent variable to investigate the role it plays in category learning (exposure time, Endress and Bonatti, 2007; Reeder et al., 2013), in nonadjacent dependency learning (speech rate, Wang et al., 2016, 2019) and in auditory statistical learning (inter-stimulus temporal distance, Emberson et al., 2011). Although these studies used different designs, stimulus materials, and forms of operationalization to the temporal variable, nevertheless, a clear pattern stands out: generally, a shorter time is beneficial to auditory rule (category) learning. However, the exact amount of time, and the mechanism and reasons for it having a positive effect on rule learning are still to be fully investigated and understood.

In order to address these gaps, this study further extends the entropy model we proposed in Radulescu et al. (2019), and puts forth an innovative information-theoretic quantification of the time-dependent variable, that is not an arbitrary manipulation of inter-stimulus temporal distance or exposure time, but the information-theoretic concept of *channel capacity* and Shannon’s noisy-channel coding theory.

## An Entropy and Channel Capacity Model for Rule Induction

Among other studies that used entropy measures to look into regularization patterns (Perfors, 2012, 2016; Ferdinand, 2015; Saldana et al., 2017; Samara et al., 2017; Ferdinand et al., 2019), Radulescu et al. (2019) and this study take a step further and propose an information-theoretic model that captures the dynamics of the interaction between the *input entropy* and the encoding capacity (*channel capacity*). This model specifies a quantitative measure for the likelihood of transitioning from encoding specific probability distributions to category formation. Specifically, our model hypothesizes that the *gradient of generalization* (Aslin and Newport, 2012) results from a *bit by bit* increase in *input entropy* per unit of time, which gradually adds up to the maximum rate of information transmission (bits/s), i.e., *channel capacity* of the learning system.

Given a random variable *X*, with *n* values {*x_1_, x_2_ … x_*n*_*}, Shannon’s entropy (Shannon, 1948), denoted by *H(X)*, is defined as:


$$H\left(X\right)=-\sum_{i=1}^{n}p\left(x_{i}\right)\log p\left(x_{i}\right){}^{1};$$

where *p(x_*i*_)* is the occurrence probability of *x*_*i*_. This quantity (H) measures the information per symbol produced by a source of input, i.e., it is a measure of the average uncertainty (or surprise) carried by a symbol produced by a source, relative to all the possible symbols (values) contained by the set (Shannon, 1948).

In Radulescu et al. (2019), in two artificial grammar experiments, we exposed adults to a three-syllable XXY artificial grammar. We designed six experimental conditions with increasing input entropy (2.8, 3.5, 4, 4.2, 4.58, and 4.8 bits). The results showed that an increase in input entropy gradually shaped *item-bound generalization* into *category-based generalization* (Radulescu et al., 2019). Thus, we obtained a precise measure of the sensitivity of a learner to the input entropy: the information load of a learner (=surprise) of the XXY structure decreases logarithmically as the input entropy increases. These findings bring strong evidence for the *gradient of generalization* depending on the probabilistic properties of the input, as proposed by Aslin and Newport (2014).

While in Radulescu et al. (2019) we probed the effect of the first factor (*input entropy*), in this study we further develop and test the model by probing the effect of the second factor – *channel capacity –* on rule induction.

### Channel Capacity in Information-Theoretic Terms

This section elaborates on the other factor of our entropy model, namely *channel capacity*, which is another information-theoretic concept in Shannon’s *noisy-channel coding theory* of a communication system. Shannon (1948) defines a communication system as having five main components: an information source (which produces a message), a transmitter (which encodes the message into a signal), a channel (the medium used to transmit the signal), a receiver (which does the inverse operation of the transmitter, that is, decodes the signal to reconstruct the message), and a destination (the person or thing for which the message is intended). In short, an information source produces a message, which is encoded by a transmitter into a signal that is suitable for transmission over a channel to a destination. The main factor under investigation here is the medium used for the transmission of information, i.e., the *channel*, and its capacity for information transmission. It follows, and it must be specified that the process of information transmission encompasses all processes starting with the transmission of information from the source to the destination, that is, all the transmission and encoding-decoding processes.

In order to define *channel capacity*, we first have to define the two main factors that are relevant for *channel capacity*: the source rate of information transmission and noise. Since the process of information transmission occurs in time, Shannon defined *source rate of information transmission* as the amount of information that a source transmits per unit of time. Information is measured using *entropy*, so *source rate of information transmission* (H′) is the amount of entropy that the source produces per unit of time (bits/s), or the source rate of information production.

The ideal case of a noiseless transmission is nearly impossible under normal real-life conditions; thus, transmission is affected by another variable, *noise*. *Noise* is defined as any random perturbations that interfere with the signal, thus rendering a *noisy channel*. The noise might perturb the signal during transmission through the channel or at either terminal end, i.e., transmitter and receiver’s end. As a result, there are missing bits of information because of a noisy transmission. Shannon (1948) defined this loss of information as *rate of equivocation (E).*

The actual rate of information transmission (R) *via* a *noisy channel* is obtained by subtracting the *rate of equivocation* (E) from the *source rate of information transmission*, H′ (Shannon, 1948):


$$R=H^{\prime }-E.$$

Note that *actual rate of information transmission* (R) is different from *source rate of information transmission* (H′), since it takes into account the loss of information due to noise (E), which occurs in the transmission of information from the source to the destination. *Source rate of information transmission* (H′) is the rate at which the source produces and transmits information, while *actual rate of information transmission* (R) is quantified at the other terminal end, i.e., the receiver, after the noise had caused a loss in information (E).

Shannon (1948) demonstrated mathematically that the capacity of *noisy channel* should be the maximum possible rate of information transmission (R), which can be obtained only if the encoding method is adequate and efficient:


$$C=Max\left(R\right)=Max\left(H^{\prime }-E\right).$$

The formula above means that the maximum rate of information transmission, i.e., *channel capacity*, can be achieved by an adequate and efficient encoding method. The efficiency of the encoding method means that the rate of equivocation (E) is kept at a minimum, in order for the actual information transmission to be as close as possible to the source rate of production. That means the received signal matches closely the sent signal, and, consequently, the message is received with the least loss of information.

According to Theorem 11 by Shannon (1948), given a certain source with a rate of information production H′ (entropy per unit of time), if H′ ≤ C, information can be sent through a noisy channel at the rate *C* with an arbitrarily small frequency of errors using a proper encoding method. If H′ > C, it is possible to find an encoding method to transmit the signal over the channel, such that the rate of equivocation is minimum, as specified by Shannon, less than H′ − C + *e* (*e* stands for errors), but the rate of transmission can never exceed C. If there is an attempt to transmit a message at a higher rate than C, using the same encoding method, then there will be an equivocation rate at least equal to the excess rate of transmission. In other words, a message can only be communicated reliably if it is encoded in such a way, i.e., using an efficient encoding method, so that the rate of information transmission, including noise, is below the capacity of the channel. In this study, we will focus on the first factor in the *channel capacity* formula, namely *source rate of information transmission.*

### Main Hypotheses of the Model About the Effect of Channel Capacity on Rule Induction

(1) *Item-bound generalization* and *category-based generalization* are outcomes of the same information encoding mechanism that *gradually* goes from a high-specificity form of encoding (*item-bound generalization)* to a more general abstract encoding (*category-based generalization)*, as triggered by the interaction between *input entropy* and the finite encoding capacity of the learning system. The encoding mechanism moves from *item-bound* to *category-based generalization* as *input entropy per unit of time* increases and becomes higher than the maximum rate of information transmission, i.e., *channel capacity*, as follows:
  (a) If the source rate of information transmission (H′–input entropy per second) is below or matches *channel capacity*, then the information can be encoded using an encoding method that matches the statistical structure of the input (the probability distribution of the specific items). Thus, if H′_  ≤ _ C, the information about specific items with their uniquely identifying (acoustic, phonological, phonotactic, prosodic, distributional, etc.) features and probability distribution (i.e., input entropy) can be encoded with a high-fidelity item specificity, and transmitted through the channel, with little loss of information, at the channel rate, the maximum rate of information transmission, and encoded by *item-bound generalization.* If H′ > C, *item-bound generalization* is impeded.
  (b) If an attempt is made to exceed the finite *channel capacity* of the encoding system, that is, the source rate of information transmission (H′–input entropy per second) does not match *channel capacity*, but it is higher than *channel capacity*, it is possible to find a proper method that encodes more information (entropy), but the rate of information transmission cannot exceed the available *channel capacity*. According to Theorem 11 (Shannon, 1948), if there is an attempt to transmit information at a rate higher than C, using the same encoding method, then there will be an equivocation rate at least equal to the excess rate of transmission. In other words, the increased source rate of information (H′ > C) brings higher inflow of *noise*, which interferes with the signal and causes an increased equivocation rate or information loss (as explained above). Thus, we hypothesize that it is precisely the *finite channel capacity* that drives the restructuring of the information, in order to find another more efficient encoding method. A more efficient encoding allows for higher input entropy per second to be encoded reliably (with the least information loss possible). As we argued in Radulescu et al. (2019), information is re-structured by (unconsciously) re-observing the item-specific features and structural properties of the input. Noise introduces random perturbations that interfere with the signal and feature configuration. This leads to instability, which unbinds features and sets them free to interact and bind into new structures. Then, similarities (shared features) that have higher significance (i.e., are “stronger” because of their higher probability) are kept in the new encoding, while differences between items (unshared features), which are insignificant features (e.g., low-probability “noisy” features) are erased or “forgotten.” This leads to a compression of the signal by reducing the number of unshared “noisy” features encoded with individual items (i.e., bits of information) and grouping them in “buckets” (categories). As a result, a new form of encoding is created, which allows for higher *input entropy* to be encoded using the available *channel capacity*, thus yielding a more general (less specific) *category-based* encoding method. Thus, *finite channel capacity* is designed to drive the re-structuring of the information for the purpose of adapting to noisier (=increasingly entropic) environments, by the principle of self-organization in line with Dynamic Systems Theory invoked in studies on other cognitive mechanisms, e.g., Stephen et al. (2009).
(2) *Channel capacity* is used here as an information-theoretic measure of the encoding capacity used in linguistic rule induction (at the computational level, in the sense of Marr (1982))^2^. In order to identify psychological correlates (at the algorithmic level), we follow experimental evidence from the *Less-is-More* hypothesis line of research, which suggests that memory constraints drive linguistic rule induction (Hudson Kam and Newport, 2005, 2009), and we embed this in classical and recent models of memory capacity and attention (Miller, 1956; Cowan, 2005; Oberauer and Hein, 2012; Baddeley et al., 2015). Hence, we hypothesize that the cognitive capacity that underlies *channel capacity*, specifically in linguistic rule induction (and, implicitly, in category formation), is the attentional capacity focused on activated representations in long-term memory, in other words working-memory capacity (WM), as defined in Cowan (2005). Rule induction can be argued to rely on the storage and online time-dependent processing capacities that support the ability to maintain active goal-relevant information (the rule), while concurrent processing (of other possible hypotheses and of noise) takes place (which is what defines WM as well, Conway et al., 2002). Corroborating evidence comes from positive correlations found between WM and domain-general categorization tasks (Lewandowsky, 2011).

Thus, while we generally deem linguistic rule induction to be supported by a domain-general WM capacity, rather than language-specific algebraic rule learning as proposed by early prominent research (Marcus et al., 1999), in this study, we are exploring specific possible memory components and WM-correlated abilities that are directly involved in linguistic rule induction (besides more general storage and retrieval components tested in previous studies under the *Less-is-More* hypothesis, Hudson Kam and Chang, 2009; Perfors, 2012). Hence, we specifically predict that one of the components underlying *channel capacity* in linguistic rule induction is a domain-general pattern recognition capacity, given that a rule induction task can be intuitively envisaged as a task of finding patterns/rules in the input.

A possible candidate test of domain-general pattern recognition is the RAVENS test (Raven et al., 2000), which was shown to be based on rule induction (Carpenter et al., 1990; Little et al., 2012) and to rely on similar storage and online time-dependent processing capacities to maintain active goal-relevant information (the rule) while concurrent processing takes place (Conway et al., 2002). Although this pattern recognition test and WM capacity are not identical (Conway et al., 2003), and apparently WM is not a causal factor for pattern recognition either (Burgoyne et al., 2019), high positive correlations were found between measures of WM capacity and tests for this domain-general pattern-recognition capacity (such as RAVENS, e.g., Conway et al., 2002; Little et al., 2014; Dehn, 2017).

## Testing the Prediction of Speeding Up the Source Bit Rate of Information Transmission

The goal of this study is to probe the effect of the time-dependent variable of the second main factor of our entropy model, *channel capacity*, on rule induction, by directly increasing *source rate of transmission* (H′), in order to attempt to exceed *channel capacity*. Theoretically, following the definition of *channel capacity* and Shannon’s Theorem 11 (Shannon, 1948), this can be achieved in two ways: either by increasing the amount of entropy (bits) at a constant rate or by speeding up the rate of feeding information (at constant bit value) into the channel. It follows that, practically, there are two methods to attempt to exceed *channel capacity*:

(1) Add stimulus-unrelated entropy (*noise*) in the input to render a noisier channel, while keeping the time variable constant. This method aims at exceeding *channel capacity* by specifically modulating the *noise* variable of *channel capacity*.
(2) Increase the source rate of information production to directly modulate the time-dependent variable of *channel capacity*. This method reduces the time that the same amount of entropy is sent through the channel, i.e., speeds up the bit rate of information transmission.

We employed the first method in another study (Radulescu et al., 2020 unpublished data), and we found that added stimulus-irrelevant entropy (*noise*) drove a higher tendency toward *category-based generalization*. In this study, we employed the second method: we increased the source rate of information transmission (*input entropy per second*) in order to directly modulate the time-dependent variable of *channel capacity*. According to our entropy model, speeding up the source rate of transmission (i.e., to a higher rate than *channel capacity*) leads to a change in encoding method, so as to avoid increased equivocation rate. Why? Because increased rate of equivocation is in fact information loss. Thus, the encoding method transitions to another encoding method in order to achieve more efficient transmission of information: that is, faster encoding rate with least information loss. Specifically, we hypothesize that increasing the source rate of information transmission leads to higher tendency to move from *item-bound* to *category-based generalization* for the purpose of achieving a more efficient encoding, with the least loss of information possible.

We tested the effect of speeding up the source rate of information transmission on both the repetition-based XXY grammar from the study of Radulescu et al. (2019) and a more complex grammar, non-adjacent-dependency grammar (aXb). The learning of a repetition-based XXY grammar requires learners to abstract away from specific items of the X and Y categories, and to move from *item-bound* to *category-based generalization*, that is, to learn a *same-same-different* rule between categories, regardless of their specific items. A source rate of transmission higher than *channel capacity* is hypothesized to boost this transition and, thus, have a positive effect on learning an XXY grammar. However, learning a non-adjacent dependency grammar is a more complex process: it entails learning item-bound dependencies between specific *a* and *b* elements and *category-based generalization* of the rich category of intervening *Xs* (Gómez, 2002; Onnis et al., 2004; Frost and Monaghan, 2016; Grama et al., 2016; Wang et al., 2019). This type of artificial grammar learning models the mechanisms needed in language acquisition to acquire rules such as *is* go-*ing, is* learn-*ing.* Thus, the learning of this type of *aXb* grammar requires learners to move from *item-bound* to *category-based generalization* for the *X* category of middle elements, while, crucially, sticking to *item-bound generalization* for specific *a_b* dependencies. If increased source rate of information transmission drives *category-based generalization* for the *X* category, it follows that it should impede *item-bound generalization* for the specific *a_b* dependencies of such an *aXb* grammar. So how does the model perform when tested on such a complex type of grammar?

Given an entropy (H) of a source and an average number of symbols produced by the source per second (*m*), we can calculate the source rate of information transmission, *H′ = mH* (Shannon, 1948). Using this formula, we estimated a source rate of transmission of information in experiments carried out by Radulescu et al. (2019). Then, we specifically predicted that, if we keep the same information content (input entropy) of the lowest entropy grammar from Radulescu et al. (2019), where there was no evidence of *category-based generalization*, but we increase the source rate of transmission up to the source rate of transmission of the highest entropy condition from the same study, where that study found high tendency toward *category-based generalization*, then we should see a higher tendency toward *category-based generalizations*, even though the statistical properties (entropy) of the input are the same.

Specifically, let us denote the source rate of information transmission in the highest entropy grammar from Radulescu et al. (2019) as H′_H_ = m_1_H_H_, and the source rate of information transmission in the lowest entropy version as H′_L_ = m_1_H_L_. Note that the average rate of symbols per second (m_1_) was the same in both versions. For the purpose of the manipulation we are aiming for, we would like to obtain H′_H_ = H′_L_ but by keeping H_L_ constant and increasing the average rate of symbols/s to obtain m_2_ such that m_2_ > m_1_. Thus, in the three-syllable XXY grammar from Radulescu et al. (2019), for a constant *m_1_ (symbols/s)*:

H_L_ = 2.8b/symbol: *H′_L_ = m_1_ H_L_*

H_H_ = 4.8b/symbol: *H′_H_ = m_1_ H_H_.*

For the purpose of increasing the source rate of transmission up to *H_H_’* while keeping entropy constant (H_L_), and by increasing the average rate of symbols/s, we calculated the necessary m_2_ as follows:

*m_2_ H_L_ = H′_H_*

*m_2_ H_L_ = m_1_ H_H_*

*m_2_/ m_1_ = H_H_ /H_L_*

*m_2_ = (4.8/2.8) m_1_*

*m_2_ = 1.71 m_1_*

Thus, we obtained *m_2_ = 1.71m_1_*, and translated it into duration of syllables and within- and between-string pauses, such that we sped up all syllables and pauses proportionally by a coefficient of 1.71. As a result, we created a faster source rate of information transmission, i.e., entropy per second (H′_L_ = H′_H_), but we kept the entropy per symbol constant H_L_ = 2.8b/symbol.

Next, for the aXb grammar, we created two versions of the grammar with different levels of entropy (H_L_; H_H_), but the same average rate of symbols/s (*m*_3_):

H_L_ = 3.52b/symbol: *H′_L_ = m_3_ H_L_*

H_H_ = 4.71b/symbol: *H′_H_ = m_3_ H_H_.*

For the purpose of increasing the source rate of information transmission up to *H′_H_* while keeping entropy constant (H_L_), and by increasing the average rate of symbols/s, we calculated the necessary m_4_ as follows:

*m_4_ H_L_ = H′_H_*

*m_4_ H_L_ = m_3_ H_H_*

*m_4_/ m_3_ = H_H_ /H_L_*

*m_4_ = (4.71/3.52) m_3_*

*m_4_ = 1.34 m_3_.*

Thus, we obtained *m_4_ = 1.34m_3_*, and translated it into duration of syllables and within- and between-string pauses, such that we sped up all elements (syllables and pauses) proportionally by a coefficient of 1.34. As a result, we created a faster source rate of information transmission, i.e., entropy per second (H′_L_ = H′_H_), but we kept the entropy per symbol constant H_L_ = 3.52b/symbol.

Besides probing the direct effect of the time variable of *channel capacity*, as presented above, this study also looked into the effect of individual differences in cognitive capacities on rule induction, to explore the cognitive capacities that underlie *channel capacity*: short-term memory capacity and a domain-general pattern-recognition capacity, as a component that reflects the working memory capacity we deem relevant for rule induction. To this end, we tested each participant on three independent tests: Forward Digit Span, as a measure of explicit short-term memory (Baddeley et al., 2015), an incidental memorization task, which measures implicit memory capacity, i.e., the ability to memorize information without being explicitly instructed to do so (Baddeley et al., 2015), and RAVENS Standard Progressive Matrices (Raven et al., 2000), which is a standardized test based on visual pattern-recognition (Carpenter et al., 1990; Little et al., 2014).

We ran two experiments to test the effect of increased rate of information on rule induction in an XXY grammar and in an aXb non-adjacent dependency grammar. Importantly, we tested the same participants in both experiments, which were conducted in two separate sessions, on two different days (at least 3 days between sessions). For practical reasons, all the participants took part first in the aXb grammar experiment (Experiment 2) and then in the XXY grammar experiment (Experiment 1). For theoretical presentation reasons, which have to do with the logic and theoretical development of the entropy model and its hypotheses, here we present the XXY experiment first, followed by the aXb experiment.

To the best of our knowledge, these are the first language learning experiments that investigate the effect of the time-dependent variable of *channel capacity* in rule induction by specifically testing information-theoretic predictions made by an entropy model.

## Experiment 1

In Experiment 1, the participants carried out three tasks. The first task presented the three-syllable XXY grammar in two different conditions: a slow source rate of information transmission (Slow Rate condition) and a fast source rate of information transmission (Fast Rate condition). In the Slow Rate condition, we used the exact stimuli and source rate of information transmission (H′_L_) as in the lowest entropy condition from Radulescu et al. (2019), 2.8 bits. In the Fast Rate condition, the same stimuli were used (H_L_ = 2.8), but the source rate of information transmission was increased by a factor of 1.71 (see section “Testing the Prediction of Speeding up the Source Bit Rate of Information Transmission”). In the test phases, the participants heard four different types of test strings (from Radulescu et al., 2019), as presented below. The participants answered a yes/no question to indicate whether the test strings could be possible in familiarization language.

Familiar-syllable XXY (XXY structure with familiar X-syllables and Y-syllables), correct answer: accept. This type of test strings probed the learning of familiar strings. Both groups were expected to accept these strings as grammatical because they were encoded as either *item-bound generalizations* (Slow Rate condition) or *category-based generalizations* (Fast Rate condition).

New-syllable XXY (XXY structure with new X-syllables and Y-syllables), correct answer: accept. This type tested whether learners moved from *item-bound* to *category-based generalization*, which enables them to accept XXY strings with new syllables. We expected that the Fast Rate group was more likely to accept these strings, as compared with the Slow Rate group. However, the absolute mean acceptance rate of these strings does not represent direct evidence for *category-based generalization*. As we argued in Radulescu et al. (2019), this rate should be compared with the mean acceptance rate of Familiar-syllable XXY strings: if the difference of the mean acceptance rate between New-syllable XXY strings and Familiar-syllable XXY strings is significantly smaller in the Fast Rate as compared with the Slow Rate condition (i.e., effect size), this would suggest that the Fast-Rate learners were more likely to have formed *category-based generalization* than the Slow-Rate learners.

Familiar-syllable X_1_X_2_Y (X_1_X_2_Y structure with familiar syllables), correct answer: reject. The participants are expected to reject these strings because the input was encoded as either *item-bound generalizations* (Slow-Rate learners) or *category-based generalizations* (Fast-Rate learners). Slow-Rate learners are expected to reject this type of strings, as their memory trace of the Familiar-syllable XXY strings is expected to be strong enough to highlight a mismatch between these strings and the Familiar-syllable X_1_X_2_Y strings. Fast-Rate learners are expected to form *category-based generalizations*, thus they should reject the Familiar-syllable X_1_X_2_Y strings as deviant from the *same-same-different* rule. However, as argued in Radulescu et al. (2019), we expect both *item-bound* and *category-based generalization* to support accuracy scores on X1X2Y strings because of different reasons: if *item-bound generalization* is developed, as (per hypothesis) learners encoded the strings as frozen *item-bound generalization*, which highlight clear mismatches between familiar and noncompliant combinations of specific items. However, memory traces of familiar items (i.e., syllables) might prompt incorrect acceptance of familiar-syllable X1X2Y. On the other side, if *category-based generalization* is fully encoded, these strings will be much more frequently rejected as non-compliant with the *same-same-different* rule, regardless of any memory trace. Thus, the higher rejection rate of these strings suggests stronger category-based encoding.

New-syllable X_1_X_2_Y (X_1_X_2_Y structure with new syllables), correct answer: reject. The participants are expected to reject this type of strings, because the input was encoded as either *item-bound generalizations* (Slow Rate group) or *category-based generalizations* (Fast Rate group).

The second task was a Forward Digit Span (Baddeley et al., 2015), and the third task was an incidental memorization task (Baddeley et al., 2015). According to the hypotheses of our entropy model, we predicted a negative effect of the explicit/incidental memory capacities on the tendency of learners to move from *item-bound* to *category-based generalization*. The rote memorization capacity (Baddeley et al., 2015) is hypothesized to have a negative effect on the transition from *item-bound* to *category-based generalization*, since a strong memory capacity for specific items and their probability configuration would support a higher *input entropy* to be encoded per unit of time (i.e., a higher *channel capacity*, in computational terms).

### Participants

Fifty-six adults, Dutch native speakers (10 males, age range 18–72, *M*_*age*_ = 26.39, *SD*_*age*_ = 11.06) participated. All the participants were naïve to the aim of the experiment, had no known language, reading, or hearing impairment or attention deficit, and received €5.

### Materials

#### Task 1: XXY Grammar

##### Familiarization Stimuli

The participants in both the Slow Rate and the Fast Rate conditions listened to the same three-syllable XXY^3^ artificial grammar used in the low entropy condition of Experiment 2 from Radulescu et al. (2019). Each string consisted of two identical syllables (XX) followed by another different syllable (Y): e.g., *ke:ke:my, da:da:li*. All syllables consisted of a consonant followed by a long vowel, to resemble common Dutch syllable structure. Seven X-syllables and seven Y-syllables were used to generate seven strings (see Supplementary Appendix A for complete stimulus set). Each string was repeated four times in each of the three familiarization phases (7 strings x 4 repetitions = 28 strings in each familiarization phase). The same 28 strings were used in all three familiarization phases, such that the entropy was the same, 2.8 bits. The participants were randomly assigned to either the Slow Rate or the Fast Rate condition, in a between-subjects design, and the presentation order of strings was randomized per participant. For entropy calculations, we employed the same method as in Radulescu et al. (2019), which is a fine-tuned extension of a related entropy calculation method proposed by Pothos (2010) for finite state grammars (see Table 1 for complete entropy calculations). In the Slow Rate condition, there was a pause of 50 ms between the syllables within strings, and a pause of 750 ms between the strings. In the Fast Rate condition, all X and Y syllables, as well as the within-and between-string pauses, were sped up separately by a factor of 1.71 using Praat (Boersma and Weenink, 2019).

**TABLE 1:** Entropy value for Experiment 1, taken from Radulescu et al. (2019).

Low entropy
H[bX] = H[7] = −Σ[0.143*log0.143] = 2.8 H[XX] = H[7] = 2.8 H[XY] = H[7] = 2.8 H[Ye] = H[7] = 2.8 H[bXX] = H[7] = 2.8 H[XXY] = H[XYe] = H[7] = 2.8 H[bigram] = 2.8 H[trigram] = 2.8 H[total] = $\frac{H\left[\text{bigram}\right]+H\left[\text{trigram}\right]}{2}$ = 2.8

##### Test Stimuli

There were three familiarization phases, interleaved with three intermediate test phases and a final (longer) test phase. Each intermediate test included four test strings, one of each type. The final test had eight test strings (two of each type): 4 + 4 + 4 + 8 = 20 test strings in total (see Supplementary Appendix A for complete stimulus set). Accuracy scores were measured as correct acceptance of Familiar-syllable XXY and New-syllable XXY strings, and correct rejection of Familiar-syllable X_1_X_2_Y and New-syllable X_1_X_2_Y strings.

We recorded all the yes/no answers and coded them as correct/incorrect answers. From all the 20 correct/incorrect answers for each participant, we calculated a proportion of correct answers per each type of test item. We performed an empirical logarithmic transformation on the proportions, to analyze the data using a linear model.

#### Task 2: Forward Digit Span

The participants were explicitly told that this was a memory test, during which a series of digits would be presented aurally, and that they would have to recall them in the same order. To prevent the participants from creating a visual pattern on the keypad while listening to the digits, we modified the standard Forward Digit Span task such that no physical keyboard was made available to the participants; rather, a row with buttons for each digit was displayed in a line on the screen only in the moment when they were asked to enter the digits by clicking the buttons, and disappeared during the listening phases. We used the standard scoring method: we measured the highest span of each participant, and recorded it as one data point per participant.

#### Task 3: Incidental Memorization Test

The participants listened to 30 bisyllabic nonsense words resembling Dutch phonology. Crucially, the participants were not told in advance that a memory test would be administered. They were only told that they were about to listen to words from another forgotten language. They were instructed to imagine what the word might have meant in the forgotten language and to pick a category (flower, animal, or tool) based on what the word sounded like to them. They had 3 s to choose a category for each word by pressing the button for flowers, animals, or tools.

After this phase, a message informed the participants that they would be given a memory test, which would check whether they remembered the words they categorized during the previous phase. They were instructed to press a yes/no button on the screen, depending on whether they have heard the word previously or not. In the memorization test, the participants gave answers on 13 targets and 13 foils. We recoded all the correct/incorrect answers into a *d’* value for each participant.

### Procedure

The participants completed the tasks in the order presented above. For Task 1, they were told that they would listen to a “forgotten language” that would not resemble any language they might know, and that the language had its own rules and grammar. The participants were informed that the language had more words than what they heard in the familiarization phases. They were told that each intermediate test would be different from the other tests, and that the tests were meant to check what they had noticed about the language. They had to decide, by pressing a Yes or a No button, if the words they heard in the tests could be possible in the language. This task lasted around 5 min. For Task 2, they were explicitly instructed that it was a memory test. For Task 3, they were not told in advance about the memory test. The entire experiment lasted for about 20 min.

### Results

**FIGURE 1:**
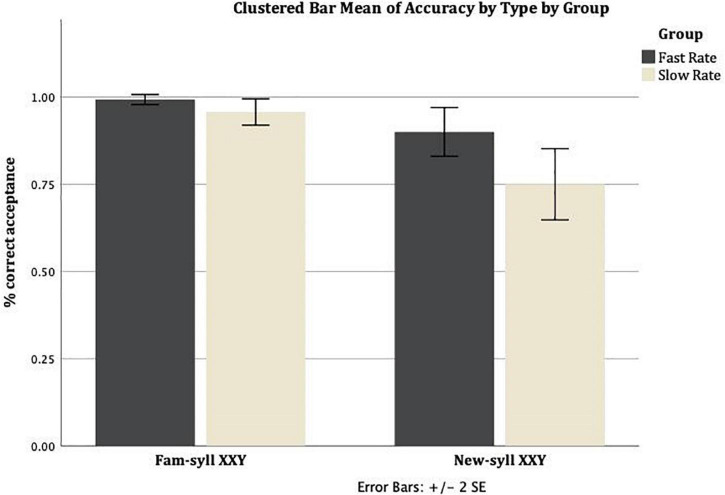
Mean rate of correct acceptance for Familiar-syllable XXY and New-syllable XXY strings in both conditions: Fast Rate and Slow Rate. Error bars show standard error of the mean.

Figure 1 presents the mean correct acceptance rate (proportion of correct acceptances per group) for Familiar-syllable XXY strings and New-syllable XXY strings, across the two conditions (Slow Rate, Fast Rate). The mean correct acceptance rate in the Slow Rate condition for Familiar-syllable XXY strings was *M* = 0.96 (*SD* = 0.1), and for New-syllable XXY strings it was *M* = 0.75 (*SD* = 0.27). The mean rate of correct acceptance in the Fast Rate condition for Familiar-syllable XXY strings was *M* = 0.99 (*SD* = 0.04), and for New-syllable XXY strings it was *M* = 0.9 (*SD* = 0.18).

**FIGURE 2:**
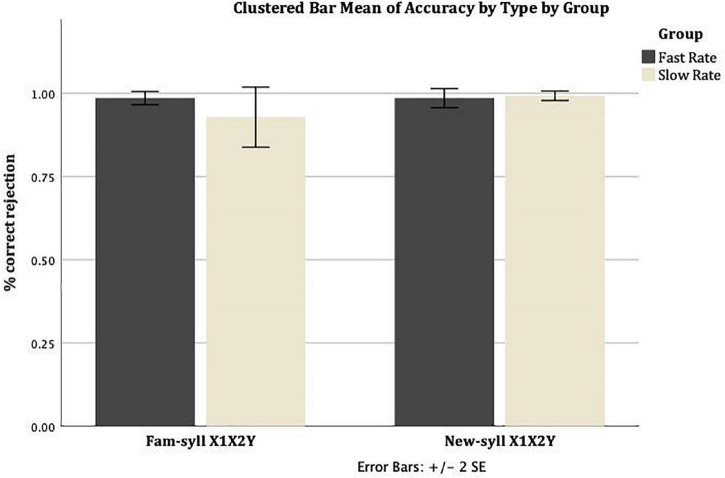
Mean rate of correct rejection for Familiar-syllable X1X2Y and New-syllable X1X2Y strings in both conditions: Fast Rate and Slow Rate. Error bars show standard error of the mean.

Similarly, Figure 2 shows the mean correct rejection rate (proportion of correct rejections per group) for Familiar-syllable X_1_X_2_Y strings and New-syllable X_1_X_2_Y strings, across the Slow Rate and Fast Rate conditions. In the Slow Rate condition, the mean correct rejection rate for Familiar-syllable X_1_X_2_Y strings was *M* = 0.93 (*SD* = 0.24), and for New-syllable X_1_X_2_Y strings it was *M* = 0.99 (*SD* = 0.04). In the Fast Rate condition, the mean correct rejection rate for Familiar-syllable X_1_X_2_Y strings was *M* = 0.99 (*SD* = 0.05), and for New-syllable X_1_X_2_Y strings it was *M* = 0.99 (*SD* = 0.08).

**FIGURE 3:**
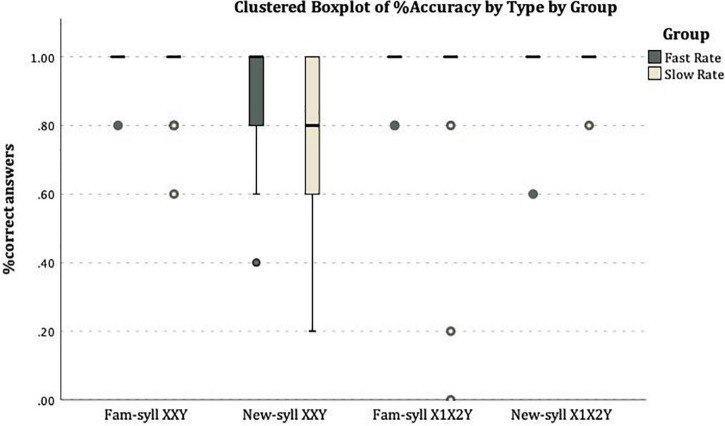
On the *X*-axis the four types of test items: Familiar-Syllable XXY, New-syllable XXY, Familiar-syllable X1X2Y, New-syllable X1X2Y. On the *Y*-axis the mean rate of correct answers: correct acceptance for XXY strings (with familiar or new syllables) and correct rejections of X1X2Y (with familiar or new syllables).

Figure 3 shows the distribution of individual mean rates per test type in both conditions.

In order to probe the effect of *channel capacity* on rule induction, we used IBM SPSS 26 to compare the performance in the two conditions (Slow Rate and Fast Rate groups) in a general linear mixed effects analysis of the relationship between Accuracy (correct acceptance of the grammatical test items and correct rejection of the ungrammatical ones) and the Rate of Transmission (Slow Rate, Fast Rate) as well as the Type of Test Strings (Familiar-syllable XXY, New-Syllable XXY, Familiar-syllable X_1_X_2_Y, New-Syllable X_1_X_2_Y). As a dependent variable, we entered Accuracy score into the model. As fixed effects, we entered Rate of Transmission, Type of Test Strings, and Rate of Transmission x Type of Test Strings interaction. As a random effect we had intercepts for subjects. The scores for Forward Digit Span, Incidental Memorization Task, and RAVENS tests^4^ were entered one by one as covariates in the model. An alpha level of.05 was used for all the statistical tests. We started fitting the data from the intercept-only model and added the random and fixed factors one by one. The model reported here is the best fitting model, both in terms of the accuracy of the model in predicting the observed data, and in terms of Akaike Information Criterion.

We found a significant main effect of Type of test strings [*F*(3,213) = 5.742, *p* = 0.001], a Rate of Transmission × Type interaction that did not reach significance [F(4,213) = 2.039, *p* = 0.09], a non-significant Forward Digit Span effect [*F*(1,213) = 0.069, *p* = 0.793], a non-significant Incidental Memorization Task effect [*F*(1,213) = 0.880, *p* = 0.349], and a non-significant RAVENS effect [*F*(1,213) = 2.326, *p* = 0.129].^5^

Pairwise comparisons of the Estimated Marginal Means (adjusted to the mean values of the covariates in the model, i.e. Forward Digit Span = 6.68, Incidental Memorization Task = 1.968, RAVENS = 71.54) revealed a significant difference between the Rate of Transmission conditions (Fast Rate and Slow Rate groups) for the New-syllable XXY [*M* = 0.101, *SE* = 0.045, *F*(1,213) = 4.936, *p* = 0.027], and a nearly significant difference for the Familiar-syllable X1X2Y [*M* = 0.085, *SE* = 0.045, *F*(1,213) = 3.522, *p* = 0.062]. For the other two Types of test, pairwise comparisons of the Estimated Marginal Means adjusted for the same level of the covariates revealed a non-significant difference between the Rate of Transmission conditions (Fast Rate and Slow Rate groups): Familiar-syllable XXY [*M* = 0.01, *SE* = 0.045, *F*(1,213) = 0.051, *p* = 0.822] and New-syllable X1X2Y [*M* = 0.012, *SE* = 0.045, *F*(1,213) = 0.069, *p* = 0.793].

Cohen’s effect size value (*d*) and the effect-size correlation (*r*) for the difference in acceptance between Familiar-syllable XXY and New-syllable XXY were higher in the Slow Rate condition (*d* = 1.03, *r* = 0.45; large effect size), than in the Fast Rate condition (*d* = 0.69, *r* = 0.32; medium effect size).

### Discussion

The results of Experiment 1 show that the mean acceptance of new XXY strings as grammatical in the familiarization language was higher in the Fast Rate condition than in the Slow Rate condition, as predicted by our model. Moreover, there was a difference between the rates of acceptance of new XXY strings vs. familiar XXY strings depending on the rate of transmission: there was a smaller difference between the mean acceptance of the new XXY strings vs. familiar XXY strings in the Fast Rate condition compared with the Slow Rate condition. This shows differences between groups in terms of how they encoded the input: if learners do not make a clear distinction between a new XXY string and a familiar XXY string, we conclude that they encoded the input as *category-based generalization*, which allows them to accept any XXY string based on the *same-same-different* rule regardless of new or familiar syllables. Hence, a smaller difference between the means of acceptance of these test types in the Fast Rate condition shows a higher tendency toward *category-based generalization* than in the Slow Rate condition. Also the rate of correct rejection of X1X2Y strings with familiar syllables was higher in the Fast Rate condition than in the Slow Rate condition, which supports the same hypothesis of our model: when speeding up the source rate of transmission, learners formed *category-based generalizations*, which helped them reject strings that violated the *same-same-different* rule, regardless of their familiar syllables. Thus, these results, together, show that there was a higher tendency toward *category-based generalization* when the source rate of transmission was increased to a rate higher than *channel capacity*, even though the input entropy was the same in both conditions, which supports the predictions of our entropy model regarding the effect of the time-dependent variable of *channel capacity* on rule induction.

We did not find a significant main effect of any of the individual differences in explicit/implicit memory capacity or RAVENS, but they improved the model as covariates. A logical possible explanation under the hypotheses of our model could be that the effect of the source rate of information was increased to such a high extent (shown by the almost at ceiling overall performance in the Fast Rate condition) that individual cognitive abilities do not make any difference. Alternatively, these particular cognitive differences do not underlie the *channel capacity* relevant for linguistic rule induction.

These results show that, even with a low input of entropy (Radulescu et al., 2019), increasing the source rate of information transmission, while controlling for individual differences in explicit/implicit memory capacity and RAVENS, drives a change in the encoding method toward a more efficient encoding. As hypothesized, the same transition to a more efficient encoding method, from *item-bound* to *category-based generalization*, was obtained by either increasing the *input entropy* (H) in Radulescu et al. (2019) or reducing the time that the same input entropy is fed into the channel, i.e., by speeding up the source bit rate of information transmission.

## Experiment 2

In Experiment 2, the participants carried out three tasks. In Task 1, the adults were exposed to an *aXb* language (Gómez, 2002; Grama et al., 2016) where they had to learn item-bound dependencies between *a* and *b* (*item-bound generalization*), while also generalizing *a_b* dependencies over a category of *X* words (*category-based generalization*). For example, they had to learn the item-bound dependency *t*ε*p_j*I*k* and generalize it over new *X* elements (like *nilbo, perxͻn*): *tεp_nilbo_j*I*k*, *tεp_perxͻn_j*I*k*, etc.

We designed two experimental conditions: a slow source rate of information transmission (Slow Rate condition) and a fast source rate of information transmission (Fast Rate condition). As presented in section “Testing the Prediction of Speeding up the Source Bit Rate of Information Transmission,” we first created two entropy versions of the grammar, with the same average rate of symbols/s (*m*_3_), then we increased the average rate of symbols/s (*m*_4_), in order to reach the same source rate of information transmission of the high entropy version while, crucially, keeping the input entropy low.

Unlike Gómez (2002), we kept *X* set size constantly high (18 *Xs*) and manipulated entropy by combining each of the three *a_b* frames with different subsets of 6 *Xs* (3 a_b* 6 *Xs*), which generated a rather low entropy grammar version (H_L_ = 3.52 bits/symbol). For the high entropy condition, the *aXb* grammar combined exhaustively each of the three *a_b* frames with all the 18 *Xs* (three a_b* 18 *Xs*), which resulted in a rather high entropy (H_H_ = 4.7 bits/symbol). Since such evaluations of low/high entropy could be seen as relative, depending on the grammar/language, we took into account previous studies on nonadjacent dependency learning (Gómez, 2002; Grama et al., 2016; Radulescu and Grama, 2020 unpublished data) in order to estimate the set size and variability necessary to achieve a low and a high entropy version. For entropy calculations, we used the same method as in Radulescu et al. (2019), see Table 2 for complete entropy calculations.

**TABLE 2:** Entropy values for the two entropy versions of the *aXb* grammar.

Low entropy	High entropy
H[begin-*a*] = H[3] = [−Σ[0.333*log0.333] = 1.58 H[aX] = H[18] = 4.17 H[Xb] = H[18] = 4.17 H[*b*-end] = H[3] = 1.58 H[begin-aX] = H[18] = 4.17 H[aXb] = H[Xb-end] = H[18] = 4.17 H[bigram] = 2.86 H[trigram] = 4.17 H[total] = $\frac{H\left[\text{bigram}\right]+H\left[\text{trigram}\right]}{2}$ = 3.52	H[begin-*a*] = H[3] = −Σ[0.333*log0.333] = 1.58 H[aX] = H[54] = 5.75 H[Xb] = H[54] = 5.75 H[*b*-end] = H[3] = 1.58 H[begin-aX] = H[54] = 5.75 H[aXb] = H[Xb-end] = H[54] = 5.75 H[bigram] = 3.67 H[trigram] = 5.75 H[total] = $\frac{H\left[\text{bigram}\right]+H\left[\text{trigram}\right]}{2}$ = 4.71

In the Slow Rate condition, we used the low entropy version as presented above H_L_ = 3.52b/symbol. In the Fast Rate condition, the same stimuli were used (H_L_ = 3.52b/symbol), but the source rate of information was sped up by a factor of (H_H_/H_L_ = 4.71/3.52 =) 1.34 (as per calculations in section “Testing the Prediction of Speeding up the Source Bit Rate of Information Transmission”).

In the test phase, the participants were asked to give grammaticality judgments on aXb strings with either correct (familiar) or incorrect (unfamiliar) *a_b* frames. Whereas familiar *a_b* frames where the same as presented during familiarization (*a_*i*__*b*_*i*_*, where *a*_*i*_ predicted *b*_*i*_ with 100% probability), unfamiliar *a_b* frames consisted of combinations between familiar a and b elements that were mismatched (*a_*i*__*b*_*j*_*, where *a* predicted another *b*). Importantly, all test strings (correct and incorrect) included new X elements that were not present in the familiarization, since we aimed at testing for generalization of non-adjacencies to new intervening elements.

Recall that, according to our entropy model, rule induction is a *phased* mechanism that moves from the first phase of *item-bound generalization* to the next-level phase of *category-based generalization* as a function of the interaction between the input entropy and *channel capacity*. Learning *aXb* strings requires both *item-bound generalization* of the *a_b* frames simultaneously with *category-based generalization* of these frames over a category of *X* elements. In this case, if the sped-up source rate of information transmission drives faster transition to *category-based generalization*, the item-bound encoding mechanism for the specific *a_b* dependencies might be phased out, and the encoding method might move to *category-based generalization* for the *a/b* elements as well, not only for the *X* category. Specifically, learners might encode the *a/b* elements as categories, which do not restrict to a specific *a_*i*__*b*_*i*_* dependency. That is, learners might not encode an *a_*i*__*b*_*i*_* relationship, but a relationship between a category of *a* elements and a category of *b* elements, which also allows for an *a_*i*__*b*_*j*_* dependency to be legit (“class-words,” Endress and Bonatti, 2007). To sum up, the predictions for this task could be opposite for the two types of relationships encoded in such an *aXb* grammar: increasing the source rate of information transmission impedes *item-bound generalization* (of the specific *a_*i*__*b*_*i*_* relationship), but it facilitates *category-based generalization* (i.e., generalizing a relationship between a/*b* categories over a category of *Xs*).

The second task that the participants had to complete was RAVENS Standard Progressive Matrices (Raven et al., 2000). According to the hypotheses of our entropy model, we predicted a positive effect of RAVENS on the tendency to move from *item-bound* to *category-based generalization*.

In the third task, the participants completed a word-recall task, designed to test item memorization, i.e., detailed phonological representations of the *a*, *b* and *X* elements, in order to test for a correlation between learners’ representations of specific items and their accuracy scores. We expected accurate memorization of the *a*/*b* elements to support better learning of the *a_b* dependencies and, thus, better accuracy scores. Conversely, failing to recall *X*s would indicate better generalization of the *X* category, hence better scores.

### Participants

The same 56 participants from Experiment 1 participated in Experiment 2. We tested one more participant in Experiment 2 (as Experiment 2 was conducted before Experiment 1, one participant did not return to participate in Experiment 1). Therefore, in total, 57 adults participated in Experiment 2 (10 males, age range 18–72, *M*_*age*_ = 26.28, *SD*_*age*_ = 11) and received €10.

### Materials

#### Task 1: aXb Grammar Learning

##### Familiarization Stimuli

All the *a* and *b* elements were monosyllabic nonsense words (e.g., *tεp, jIk*), while all the *X* elements were bisyllabic nonsense words (e.g., *naspu, dyfo:*), based on Grama et al. (2016). Each *a_b* pair was combined with a different, non-overlapping set of six *X* elements (see Supplementary Appendix B for the complete stimulus set). In both Slow Rate and Fast Rate conditions, two versions of the *aXb* language were used: Language 1 (L1) and Language 2 (L2). The only difference between L1 and L2 was the specific legit combination of the three *a* and *b* elements into pairs: *tεp _lœt, sͻt_ jIk*, and *rak_tuf* (L1), and *tεp _ jIk, sͻt_tuf*, and *rak_lœt* (L2). Therefore, every *a_*i*__*b*_*i*_* pair in L1 was ungrammatical (*a_*i*__*b*_*j*_*) in L2, and vice versa. We used two different versions to prevent an effect of idiosyncrasies of particular *a_b* combinations (L1 or L2). Therefore, each version of the *aXb* grammar (L1 and L2) consisted of (3 *a_*i*__*b*_*i*_* ^∗^ 6 X_*i*_ =) 18 different *a_*i*_X_*i*_b_*i*_* strings. Each participant listened to only one version of the *aXb* grammar (either L1 or L2), and to only one source rate of transmission condition (either Slow Rate or Fast Rate).

The 18 different *a_*i*_X_*i*_b_*i*_* strings were presented 12 times, resulting in a total of 216 strings, in a randomized order for each participant. In the Slow Rate condition, there was a 100-ms within-string pause, and a 750-ms between-string pause. In the Fast Rate condition, all the *a, b*, and *X* elements, as well as the within-string and between-string pauses for each *aXb* string, were sped up by a factor of 1.34 (see section “Testing the Prediction of Speeding up the Source Bit Rate of Information Transmission”) using Praat (Boersma and Weenink, 2019). The duration of each *a, b*, and *X* word was shortened separately by the 1.34 factor, and then the elements were spliced into the specific *aXb* strings.

##### Test Stimuli

Each *a_b* frame of each language (L1 and L2) was combined with two novel *X* elements to yield (6 *a_b* ^∗^ 2 *X* =) 12 new test items (see Supplementary Appendix B). Each participant listened to 12 new *aXb* strings: six grammatical and six ungrammatical. The six new *aXb* strings that contained the L1 *a_b* pairs were counted as ungrammatical for the L2 learners, while the six new *aXb* strings with the L2 *a_b* pairs were ungrammatical for the L1 learners. Accuracy scores for learning the *aXb* grammar were calculated as correct acceptances of the grammatical strings and correct rejections of the ungrammatical strings.

#### Task 2: RAVENS

The second task was Raven’s Standard Progressive Matrices (Raven et al., 2000), for which the participants had to solve 60 matrices by identifying which pattern is missing in a multiple choice task. Each matrix consists of a set of nine patterns, of which one is missing, arranged in a particular order according to some underlying rules. The standard RAVENS allows 50 min for completion, but after a pilot, we allowed the participants only 35 min, to avoid a time-consuming and exhausting experiment session. We used the standard scoring method: we counted all correct answers, and then we used the standard tables to transform them into age-corrected percentiles.

#### Task 3: Word Recall Task

The Word Recall task had two tests. In the first test, the participants were presented visually with 12 familiar two-syllable *X* words from the *aXb* language, and 12 new bisyllabic foils, similar to the familiar ones, which overlapped in one syllable with the target words. The second test presented the participants visually with six monosyllabic familiar *a* or *b* elements of the *aXb* language, and six new nonsense word foils, which differed from the target words only by one letter (see Supplementary Appendix C for stimulus set). The participants had to indicate for each word whether they heard it during the first task. Accuracy scores were measured as correct acceptances of the familiar items and correct rejections of the foils.

### Procedure

Before the familiarization phase of Task 1, the participants were instructed that they would listen to an “alien language” that does not resemble any language that they might be familiar with, and that the language has its own rules and grammar. To avoid any motivation to explicitly look for patterns in the stimuli, the participants were not informed of the subsequent test phase until after the end of the familiarization phase. Before the test phase, the participants were instructed that they would listen to new sentences in the same “alien language,” and that none would be identical to the sentences they had heard before. They were then asked to decide for each sentence whether it was correct or not, according to the grammar of the language they had just heard, by clicking on “Yes” or “No.” They were instructed to answer quickly and intuitively. Afterward, the other tasks were administered in the order stated above. Experiment 2 lasted approximately 1 h.

### Results

Table 3 shows the means and standard deviations of accuracy scores (proportion correct responses) for both conditions (Slow Rate vs. Fast Rate).

**TABLE 3:** Descriptive statistics of mean correct score in two conditions of exposure. Experiment 2.

Condition	*M*	*SD*	*n*	*SE*	*95% CI for Mean Difference*
Slow rate	0.69	0.46	29	0.09	0.51, 0.87
Fast rate	0.55	0.50	28	0.09	0.37, 0.74

**FIGURE 4:**
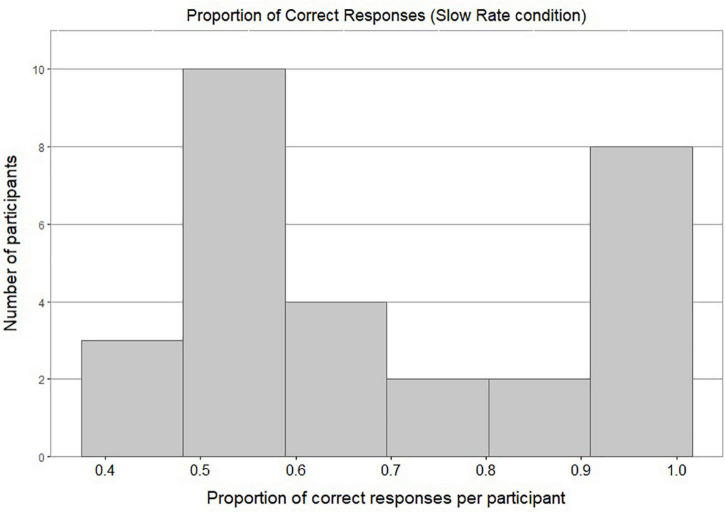
Histogram of proportion of correct responses per participant in Slow Rate Condition.

**FIGURE 5:**
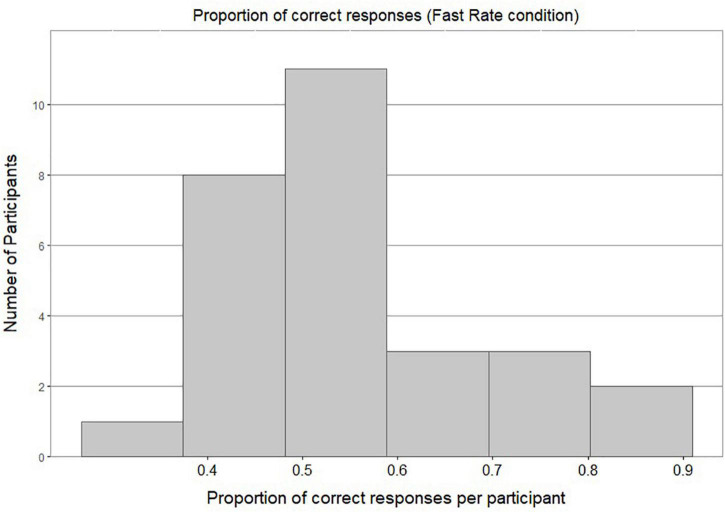
Histogram of proportion of correct responses per participant in Fast Rate Condition.

Figure 4 shows a bimodal distribution of individual accuracy scores in the Slow Rate condition: this shows that most of the participants either performed around chance level or achieved a very high accuracy score. Figure 5 shows most of the participants in the Fast Rate condition performed between 40 and 60%.

Because the data were not normally distributed, a nonparametric statistical test, a two-tailed one-sample Wilcoxon signed-rank test, was conducted to assess whether response rates were significantly different from chance. The accuracy score of Fast-Rate learners (*M* = 0.55, *SD* = 0.5) was significantly different from chance at the 0.05 level of significance, with a moderate effect size (*p* = 0.017, 95% CI for mean difference 0.5 to 0.63, *r* = 0.45). The accuracy score of Slow-Rate learners (*M* = 0.69, *SD* = 0.46) was significantly different from chance at the 0.05 level of significance, with a large effect size (*p* < 0.001, 95% CI for mean difference 0.67 to 0.83, *r* = 0.73).

To compare performance across the two conditions, we used R (R Core Team, 2017) and the lmerTest package (Kuznetsova et al., 2017) to perform a general linear mixed effects analysis of the relationship between Accuracy (correct acceptance of grammatical test strings and correct rejection of ungrammatical test strings) and Rate of Transmission (Slow Rate, Fast Rate). As a dependent variable, we entered Accuracy in the model, and as fixed effects we entered Rate of Transmission (Slow Rate, Fast Rate) and Language (L1, L2), without interaction term. As random effects we had intercepts for subjects^6^. An alpha level of 0.05 was used for all the statistical tests. We started fitting the data from the intercept-only model and added the random and fixed factors one by one. The model reported here is the best fitting model, both in terms of model accuracy in predicting the observed data and Akaike Information Criterion. Likelihood Ratio Tests were performed separately as a means to attain *p*-value for the effect of each predictor (Rate of Transmission, Language).

A significant main effect of Rate of Transmission [χ^2^(1) = 8.43, *p* = 0.003, conditional *R*^2^ = 0.1] on Accuracy was found, indicating that the participants in the Fast Rate condition had significantly lower Accuracy scores as compared with the participants in the Slow Rate condition. Language was not a significant predictor [χ^2^(1) = 3.2, *p* = 0.07, conditional *R*^2^ = 0.09]. Finally, we ran an additional model that included the interaction between Rate of Transmission and Language (although this was not the best fitting model, we wanted to verify that our specific stimuli did not prompt different performance). No significant interaction effect was found between Rate of Transmission and Language [χ^2^(1) = 0.14, *p* = 0.7, conditional *R*^2^ = 0.1]. The scores of individual differences tests (Forward Digit Span, Incidental Memorization Test, Raven’s Progressive Matrices, Word Recall Test) were added to this model as fixed factors, one by one. However, the only one that improved the model was the accuracy score in the Word Recall Test for *a*/*b* (but not *X*) elements of the *aXb* grammar, and it also had a significant positive effect on the Accuracy scores [χ^2^(1) = 3.8, *p* = 0.05, conditional *R*^2^ = 0.1].

### Discussion

In Experiment 2, we tested the effect of speeding up the source rate of transmission on learning a complex *aXb* grammar, which required both *item-bound generalization* of the specific *a_b* dependencies and *category-based generalization* in order to generalize those dependencies over a category of intervening *X* elements. According to our entropy model, our predictions for this experiment were opposite for the two types of relationships encoded in an *a_*i*_Xb_*i*_* grammar: increasing the source rate of information transmission impedes *item-bound generalization* (of the specific *a_*i*__*b*_*i*_* relationship), but it facilitates *category-based generalization* (i.e., generalizing a relationship between *a* and *b* categories over a category of *Xs*). The results showed that there was indeed a significant effect of increasing the source rate of transmission on learning the *aXb* grammar, such that the Fast Rate group scored lower than the Slow Rate group. This shows that increasing the source rate of transmission by a factor of 1.34 in this particular *a_*i*_Xb_*i*_* grammar with an entropy of 3.52 bits/symbol makes learning of the specific *a_*i*__*b*_*i*_* frames and generalizing them over novel intervening *X* elements more difficult than a slower rate of transmission. Moreover, participants who recalled the *a/b* elements better across conditions learned the specific *a_*i*__*b*_*i*_* frames better. Thus, the learning of *a_*i*_Xb_*i*_* grammar is correlated with item-specific encoding of the *a/b* elements. All these results, taken together, support the predictions of our entropy model, namely, that an increased source rate of information transmission impedes *item-bound generalization* (of the specific *a_*i*__*b*_*i*_* relationship).

**FIGURE 6:**
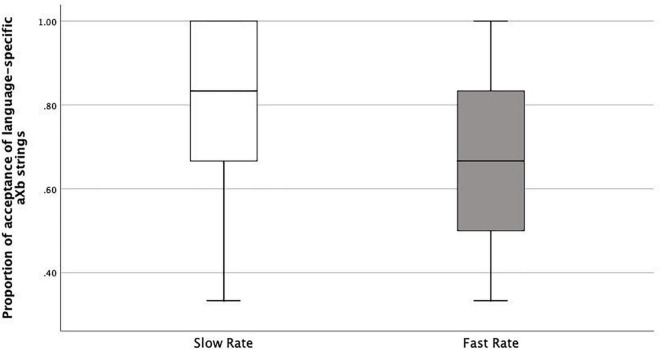
Boxplots of proportions of acceptance of language-specific aXb strings in Slow Rate Condition as compared to Fast Rate Condition.

**FIGURE 7:**
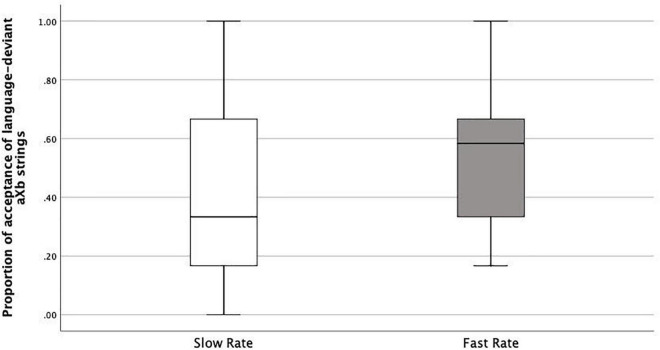
Boxplots of proportions of acceptance of language-deviant aXb strings in Slow Rate Condition as compared to Fast Rate Condition.

As we argued above, if learners correctly accept new *aXb* strings with the specific familiar *a_*i*__*b*_*i*_* dependencies and new *X* elements, it shows they were both able to encode *item-bound generalizations* (*a_*i*__*b*_*i*_* frames), and to generalize them over a category of *X* elements, i.e., *category-based generalization*. This is what happened both in the Slow Rate and Fast Rate conditions. However, the Fast Rate group had a lower tendency to do so compared with the Slow Rate group. There could be several logical interpretations: Fast-Rate learners failed at *category-based generalization* of the *Xs*, they failed at *item-bound generalization* of the *a_*i*__*b*_*i*_* frames, or they were simply confused. Therefore, we looked into the acceptance/rejection ratios. If the first case was true, rejection rates should be higher than acceptance rates, since all the test items had new *Xs*. This was not the case. Actually, the Fast-Rate learners show similarly high acceptance rates for both language-specific *a_*i*_Xb_*i*_* strings (specific to the exposure language, e.g., L1) and language-deviant *a_*i*_Xb_*j*_* strings (specific to the other language, e.g., L2), with a rather high acceptance rate for the language-deviant *a_*i*_Xb_*j*_* strings (Median = 0.58) compared with the Slow-Rate learners (median = 0.33) (Figures 6, 7). This points to the fact that the Fast-Rate learners failed to learn the specific *a_*i*__*b*_*i*_* dependencies, that is, *item-bound generalization* was impaired in the Fast Rate group.

If this was the case, this result can be accounted for by our entropy model: as we argued in section “Experiment 2”, a sped up source rate of information transmission precipitates the transition to *category-based generalization* faster, such that the item-bound encoding mechanism for the specific *a_*i*__*b*_*i*_* frames might be phased out, and the encoding method moves to *category-based generalization* for the *a_*i*__*b*_*i*_* frames as well. This would be a case of overgeneralization: categories of the *a*/*b* elements would be inferred (i.e., *category-based generalization*), not just the item-bound specific *a_*i*__*b*_*i*_* frames, so any *a* could freely combine with any *b*, such that the *a_*i*__*b*_*j*_* frames would also be accepted (“class-words”). Since all the test items show new combinations with *X* elements, the learner might find it highly probable that the *a/b* elements could yield new combinations, as long as they preserve the main *aXb* order and word characteristics (i.e., monosyllabic *a* followed by a bisyllabic *X* and then a monosyllabic *b*).

Following this logic, if the Fast-Rate learners actually overgeneralized, they must have started the test by accepting both language-specific and language-deviant *aXb* strings, and after the first acceptances they would question why all the test items seem to be acceptable, which might have led to an increased rate of rejections in the last part of the test. Alternatively, if the Fast-Rate learners were just confused, the acceptances should be randomly scattered over test trials.

An inspection of the acceptance rate of both language-specific and language-deviant *aXb* strings, in the Fast Rate condition, showed a higher tendency to accept all the test strings in the first three trials of the test [*t(11)* = −1.951, *p* = 0.05], regardless of exposure language, than in the last trials. These results might point to a case of overgeneralization in the Fast Rate condition.

Thus, it is possible that the source rate of information transmission was increased to an extent higher than required to actually learn the *a_*i*_Xb_*i*_* grammar, and that it led to overgeneralization. Further research should specifically test the overgeneralization hypothesis, and look further into the effect of sped-up source rate of information transmission at a lower rate, i.e., a speeding up factor *m < 1.34*, to find the adequate source rate of transmission for learning this complex grammar.

## General Discussion and Conclusion

This article contributes to the ongoing research on the underlying mechanisms and factors that drive both *item-bound generalization* and *category-based generalization* by extending further the entropy model for rule induction that we proposed in Radulescu et al. (2019). Our entropy model offers a more refined formal approach to the classical *Less-is-More* hypothesis (Newport, 1990) and takes a step further by bringing together two factors in one information-theoretic account based on Shannon’s noisy-channel coding theory (Shannon, 1948). Specifically, our model hypothesizes that an increase in the source *input entropy per second* to a rate higher than the time-sensitive encoding capacity of our brain, *channel capacity*, drives the transition from *item-bound* to *category-based generalization*. In Radulescu et al. (2019), in two artificial grammar experiments, we found evidence that an increase in input entropy gradually shapes *item-bound generalization* into *category-based generalization*. Hence, our model specifically predicts that it is not high entropy in absolute terms that is the factor at stake in this mechanism. Rather, our finite entropy-processing *channel capacity*, places an upper bound on the amount of entropy per second, which drives the self-organization of information from an encoding method to another, in line with Dynamic Systems Theory (Stephen et al., 2009).

In two artificial grammar experiments, an XXY grammar and a more complex aXb grammar, we sped up the source rate of information transmission to tax *channel capacity*, which was hypothesized to drive the transition from *item-bound* to *category-based generalization*. Learning an XXY grammar requires abstracting away from specific items of the X and Y categories, to move from *item-bound* to *category-based generalization*, that is, to learn the *same-same-different* rule between categories, regardless of specific items. The results showed that this transition was driven by an increase in the source rate of information transmission, i.e., *input entropy per second*, while the statistical properties of the input, i.e., *input entropy per symbol*, remained constant at a low level, which did not support the generalization in Radulescu et al. (2019). Crucially, as hypothesized by our entropy model, moving from *item-bound* to *category-based generalization* was driven by either increasing the *input entropy* (H) in Radulescu et al. (2019) or increasing the time that the same input entropy enters the channel, thus, taxing the *channel capacity* in this study.

Learning an *aXb* grammar requires moving from *item-bound* to *category-based generalization* for the category of middle *Xs*, while, crucially, sticking to *item-bound generalization* for the specific *a_b* dependencies. If increased source rate of information transmission drives *category-based generalization* for the *X* category, it follows that it should phase out *item-bound generalization* for the specific *a_b* dependencies. Indeed, the results showed that faster source rate of information caused lower accuracy than slower source rate of information on this grammar. As per our model, one logical interpretation of these results would be that the source rate of transmission was too high for this type of grammar with the specific input entropy that we tested (3.52 bits), and that it precipitated the transition to *category-based generalization* for the specific *a_b* dependencies as well, not only for the *X* elements. This points to a possible overgeneralization, where learners might have learned an *AXB* grammar, where A and B stand for categories instead of *item-bound* relationships between specific *a*/*b* elements. Indeed, it is possible that for this type of grammar fast, but not furious, might yield better learning. Future research should look into a slower rate of transmission for an *aXb* grammar with this specific entropy (3.52 bits).

Altogether, these results show that, as hypothesized by our entropy model, rule induction is an encoding mechanism that moves from *item-bound* to *category-based generalization* driven by the interaction between the *input entropy* and the finite *channel capacity.* Future research should look into the exact mathematical relationship between input entropy and rate of transmission, by also considering the other variable of *channel capacity*, i.e., the rate of equivocation caused by noise interference, in order to calculate an estimation of the *channel capacity* for rule induction.

Although having used other methods than information-theoretic approaches to investigate the effect of a time-dependent variable on category learning (Reeder et al., 2009, 2013), on non-adjacent dependency learning (Endress and Bonatti, 2007; Wang et al., 2016, 2019) and on auditory statistical learning (Emberson et al., 2011), converging evidence from these studies highlights a clear pattern: generally a shorter time is beneficial to auditory rule (category) learning. This hypothesis is also supported by evidence from neural network research showing that reduced training time leads to lower generalization error (Hardt et al., 2016). Our study contributes to this research topic by taking a step further: it applies a purely information-theoretic measure directly derived from Shannon’s noisy-channel coding theory and based on the quantified amount of input entropy per second.

Our model is compatible with another information-theoretic hypothesis derived from Shannon’s noisy-channel coding theory: the hypothesis of Uniform Information Density (Jaeger, 2006, 2010; Levy and Jaeger, 2007). Although proposed in a different domain of application, this hypothesis proposes that in language production speakers prefer (intuitively) to encode their message by a uniform distribution of information across the signal, with a rate of information transfer close to the channel capacity, but without exceeding it. In other words, language production is inherently a mechanism designed for efficient communication, in that it balances the amount of information per time or signal (dubbed “information density”), such that the channel is never under- or overutilized (Jaeger, 2010). Underutilization means a waste of channel, while overutilization risks information loss, as per Shannon’s noisy-channel coding theory, hence, as per the Uniform Information Density. By posing the noisy-channel capacity as an upper bound of the rate of information transmission for the purpose of efficient transmission without information loss, our model accounts for the Uniform Information Density hypothesis, and takes a step further by offering a more general domain of application (i.e., learning and generalization).

At the algorithmic level (in the sense of Marr, 1982), our entropy and channel capacity model for rule induction in artificial grammar is compatible with recent models of recognition memory (Cox and Shiffrin, 2017) and exemplar models applied to artificial grammar learning (Jamieson and Mewhort, 2010). Future research should look into the link between our entropy model and these formal approaches based on encoding instances as vectors of features, with generalization being triggered by vector similarity (Chubala and Jamieson, 2013). Indeed, as we argued in Radulescu et al. (2019), by refining the feature similarity approach to the category formation proposed by Aslin and Newport (2012, 2014), our entropy model suggests that information is re-structured from *item-bound* to *category-based generalization* by (unconsciously) re-observing the structural properties of the input and identifying similarities (shared features) and specific differences (unshared features) between items. Crucially, our model proposes *channel capacity* as the upper bound on the amount of similarities/differences encoded. The degree of specificity of the encoding (i.e., *item-bound* specificity) is given by the amount of differences encoded with specific items, which results from a lower or higher *input entropy* (measured in bits of information): the more differences are encoded (higher *input entropy*), the higher the degree of specificity of the encoding (i.e., *item-bound generalization*). Conversely, when the degree of specificity of the encoding reaches the upper bound placed by *channel capacity* on the number of bits encoded per second, a reduction or “gradual forgetting” of the encoded differences is triggered in order to avoid an inefficient, i.e., noisy, encoding (Radulescu et al., 2019). Hence, more and more similarities between items are highlighted, which drives an automatic gradual grouping of items under the same “bucket.” Hence, the degree of specificity decreases and the degree of generality increases *gradually* with each bit of information. Thus, a gradient of specificity/generality on a continuum from *item-bound* to *category-based generalizations* can be envisaged in terms of number of bits of information encoded in the representation (analogous to the degree of stability/plasticity in terms of strength of memory pathways in neural networks, Abraham and Robins, 2005).

A follow-up topic would be to better define and specify *channel*, be it a communication channel between speakers or an abstract channel as we mostly hinted in this study: an abstract channel between an abstract source, a grammar, and a learner. However, we would briefly suggest a more in-depth and granular understanding of the abstract concept of *channel* as a system of channels: intuitively, and oversimplifying here, the acoustic signal from the environment enters the acoustic channel of a learner, which has a specific rate of information transmission, then the output of this channel becomes the input to the perception channel, whose output becomes the input to the cognitive channel. Estimates of the bit rate of information processing by applying information theory were proposed in some perception and cognitive domains, e.g., in visual attention (Verghese and Pelli, 1992), visual processing (Koch et al., 2006), unconscious vs. conscious processing (Dijksterhuis and Nordgren, 2006), and cognitive control (Wu et al., 2016). However, we suggest that the concept of *channel* should be first and foremost defined and specified in physical and biological terms (i.e., at the level of brain structure and neural networks), and further investigated in terms of its link to the cognitive capacities (at the algorithmic level). That would mean further investigating and applying Shannon’s *channel* and *noisy-channel coding theory* to recent developments in neurobiology, where it was shown that artificially induced forgetting at the cellular level drives generalization (Migues et al., 2016). Moreover, since information is physical (Laughlin et al., 1998; Machta, 1999; Karnani et al., 2009), further research should look into the information-theoretic concept of *channel* and *rate of information transmission* at the level of neural networks. Neural networks are the physical/biological medium (i.e., channel) transmitting one form of information (acoustic energy) to the brain that is transcoded into another form of information (i.e., neuronal energy, patterns of electric activity at the neuronal level). Physical bioprocesses of energy transformation from acoustic information into electric signal and transmission through neural networks were proposed to underlie abstract memory representations (Varpula et al., 2013).

Before concluding, it is imperative to clarify one aspect. A model of *finite* and *noisy-*channel capacity might lead the reader to assume a kind of a cognitive limitation as in a flaw of the cognitive system, which is definitely not the case. We do not propose a model in which the emergence of rules and categories, i.e., structure, is merely the side effect of some constraints of a limited biological system. In accordance with innovative theories and findings in neurobiology (Frankland et al., 2013; Hardt et al., 2013; Migues et al., 2016; Richards and Frankland, 2017), we deem our *finite* and *noisy-*channel capacity to be a design feature of our biological system for adaptive purposes. More precisely, neurobiological evidence shows that our memory system is designed to encode memories not as in-detail representations of the past, but as simplified models better suited for future generalization in noisy environments (Richards and Frankland, 2017). The brain employs several strategies to undermine faithful in-detail representations to prevent overfitting to past events (in accordance with neural networks research (MacKay, 2003; Hawkins, 2004), which promotes better generalization (among which is *noise* injection, a neurobiological mechanism that increases random variability in the synaptic connections, Villarreal et al., 2002).

Fast but not furious, reads the title of this article. Speed up, but not wildly and in an unrestrained fashion. The channel capacity acts as a speedometer, and determines the maximum rate of information transmission with adequate encoding. In this study, we proposed an innovative method to increase the rate of information to tax *channel capacity*. We found that increasing the rate of transmission with a specific factor calculated by applying Shannon’s formula to experimentally obtained data indeed has the hypothesized effect on rule learning: it drives *category-based generalization*, and it interferes with *item-bound generalization*. Thus, we deem it necessary to specify that by sped-up bit rate we do not mean that an unrestrained increased bit rate, in absolute terms, up to very high bit rates, drives rule induction in any context, or grammar. In other words, the very specific dynamics between the *input entropy* and the maximum *rate of information transmission* drive rule induction. Further research should investigate this sweet spot and find the mathematical relationship between these two factors.

## Data Availability Statement

The raw data supporting the conclusions of this article will be made available by the authors, without undue reservation.

## Ethics Statement

The studies involving human participants were reviewed and approved by Ethics Assessment Committee Linguistics (ETCL), Utrecht Institute of Linguistics OTS. The patients/participants provided their written informed consent to participate in this study.

## Author Contributions

SR developed the entropy model and the idea for the study (with input from SA and FW). SR and IG designed the experiments. SR, IG, and AK created the materials. AK recruited and tested the participants. AK and SR analyzed the data. AK wrote a shorter preliminary draft as her internship report. SR wrote the publishable manuscript with input from IG, FW, SA, and AK. All the authors contributed to the article and approved the submitted version.

## Conflict of Interest

The authors declare that the research was conducted in the absence of any commercial or financial relationships that could be construed as a potential conflict of interest.

## Publisher’s Note

All claims expressed in this article are solely those of the authors and do not necessarily represent those of their affiliated organizations, or those of the publisher, the editors and the reviewers. Any product that may be evaluated in this article, or claim that may be made by its manufacturer, is not guaranteed or endorsed by the publisher.

## Footnotes

2 Although with different definitions and applications, *channel capacity* has previously been used in an early study on capacity in memory studies on psychology (Miller, 1956) and in more recent mathematical modeling for inferring workload capacity using response time hazard functions (Townsend and Ashby, 1978; Townsend and Eidels, 2011).

3 Each letter stands for a set of syllables that do not overlap, that is the subset of X-syllables does not overlap with the subset of Y-syllables.

4 RAVENS scores were obtained for the participants during the second experiment presented in this paper, since the same participants participated in both experiments (see section “Experiment 2” below).

5 We also checked the main effect of Rate of Transmission, and since it was non-significant [*F*(1,213) = 2.558, *p* = 0.111], it did not improve the model, and it created effects of an overfitted model, we excluded it from the final model presented here.

6 Due to convergence issues, random intercepts for items were excluded due to convergence issues [their estimated variance was zero, they did not improve the model and their effect was insignificant – χ^2^(1) = 0, *p* = 1)].
