# Supplementary material for: Fast but Not Furious. When Sped Up Bit Rate of Information Drives Rule Induction
Source: Front Psychol. 2021 Nov 11;12:661785. doi: 10.3389/fpsyg.2021.661785 (PMC8632011; doi:10.3389/fpsyg.2021.661785)
Supplement: Supplementary file 1 [file Table_1.pdf]

Appendix A

Familiarization strings
ke:ke:my
jujuɣo
da:da:li
pypyve:
tø:tø:rø:
hihisa:
fofoʃu
ke:ke:my
jujuɣo
da:da:li
pypyve:
tø:tø:rø:
hihisa:
fofoʃu
ke:ke:my
jujuɣo
da:da:li
pypyve:
tø:tø:rø:
hihisa:
fofoʃu
ke:ke:my
jujuɣo
da:da:li
pypyve:
tø:tø:rø:
hihisa:
fofoʃu

Test strings

Type	Test 1	Test 2	Test 3	Final Test	
Familiar-syllable XXY	da:da:li	hihisa:	ke:ke:my	tø:tø:rø:	jujuɣo
New-syllable X1X2Y	poxa:ru	runyni	xa:misy	syniny	mininy
New-syllable XXY	dydyta:	zuzuvo	sosory	jijifø:	ʋuʋuse:
Familiar-syllable X1X2Y	juda:sa:	pytø:my	ke:fove:	hida:rø:	tø:pyɣo

Appendix B

	a/b	***IPA***
a1	tep	[tɛp]
a2	sot	[sɔt]
a3	rak	[rɑk]
b1	lut	[lœt]
b2	jik	[jik]
b3	toef	[tuf]
	X	
**No.**	***Familiarization***	***IPA***
1	blieker	[blikər]
2	dufo	[dyfo]
3	fidang	[fidɑŋ]
4	gopem	[xopəm]
5	kengel	[kɛŋəl]
6	kijbog	[kɛibɔx]
7	loga	[loxa]
8	malon	[malɔn]
9	movig	[movix]
10	naspu	[nɑspu]
11	nijfoe	[nɛifu]
12	noeba	[nuba]
13	plizet	[plizɛt]
14	rajee	[raje]
15	rogges	[rɔxəs]
16	seeta	[seta]
17	snigger	[snixər]
18	wabo	[vɑbo]
	Test (novel Xs)	
19	nilbo	[nilbo]
20	pergon	[perxɔn]

Appendix C

Word Recall X

Target X word used	*X word not used*	*Foil*
[movix]		
[vɑbo]		
[nɛifu]		
[seta]		
[kɛŋəl]		
[rɔxəs]		
[dyfo]		
[nuba]		
[kɛibɔx]		
[snixər]		
[fidɑŋ]		
[plizɛt]		
	[nɑspu]	[nɑsfu]
		[lɛipu]
	[xopəm]	[xobər]
		[vapəm]
	[raje]	[rafo]

		[poje]
	[blikər]	[blifot]
		[prukər]
	[loxa]	[lopεi]
		[pixa]
	[malɔn]	[mazət]
		[silɔn]

Word Recall a/b

Target	*Foil*
[tεp]	[fεp]
[sɔt]	[sɔs]
[rɑk]	[rɑuk]
[lyt]	[lym]
[jik]	[juk]
[tuf]	[xuf]
